# Genome-Wide Association Analyses Identify *SPOCK* as a Key Novel Gene Underlying Age at Menarche

**DOI:** 10.1371/journal.pgen.1000420

**Published:** 2009-03-13

**Authors:** Yao-Zhong Liu, Yan-Fang Guo, Liang Wang, Li-Jun Tan, Xiao-Gang Liu, Yu-Fang Pei, Han Yan, Dong-Hai Xiong, Fei-Yan Deng, Na Yu, Yin-Ping Zhang, Lei Zhang, Shu-Feng Lei, Xiang-Ding Chen, Hong-Bin Liu, Xue-Zhen Zhu, Shawn Levy, Christopher J. Papasian, Betty M. Drees, James J. Hamilton, Robert R. Recker, Hong-Wen Deng

**Affiliations:** 1 School of Medicine, University of Missouri Kansas City, Kansas City, Missouri, United States of America; 2 Institute of Molecular Genetics, School of Life Science and Technology, Xi'an Jiaotong University, Xi'an, People's Republic of China; 3 Laboratory of Molecular and Statistical Genetics, College of Life Sciences, Hunan Normal University, Changsha, Hunan, People's Republic of China; 4 Osteoporosis Research Center, Creighton University, Omaha, Nebraska, United States of America; 5 Vanderbilt Microarray Shared Resource, Vanderbilt University, Nashville, Tennessee, United States of America; The Wellcome Trust Centre for Human Genetics, University of Oxford, United Kingdom

## Abstract

For females, menarche is a most significant physiological event. Age at menarche (AAM) is a trait with high genetic determination and is associated with major complex diseases in women. However, specific genes for AAM variation are largely unknown. To identify genetic factors underlying AAM variation, a genome-wide association study (GWAS) examining about 380,000 SNPs was conducted in 477 Caucasian women. A follow-up replication study was performed to validate our major GWAS findings using two independent Caucasian cohorts with 854 siblings and 762 unrelated subjects, respectively, and one Chinese cohort of 1,387 unrelated subjects—all females. Our GWAS identified a novel gene, *SPOCK* (Sparc/Osteonectin, CWCV, and Kazal-like domains proteoglycan), which had seven SNPs associated with AAM with genome-wide false discovery rate (FDR) *q*<0.05. Six most significant SNPs of the gene were selected for validation in three independent replication cohorts. All of the six SNPs were replicated in at least one cohort. In particular, SNPs *rs13357391* and *rs1859345* were replicated both within and across different ethnic groups in all three cohorts, with *p* values of 5.09×10^−3^ and 4.37×10^−3^, respectively, in the Chinese cohort and combined *p* values (obtained by Fisher's method) of 5.19×10^−5^ and 1.02×10^−4^, respectively, in all three replication cohorts. Interestingly, *SPOCK* can inhibit activation of *MMP-2* (matrix metalloproteinase-2), a key factor promoting endometrial menstrual breakdown and onset of menstrual bleeding. Our findings, together with the functional relevance, strongly supported that the *SPOCK* gene underlies variation of AAM.

## Author Summary

Menarche is a physical milestone in a woman's life. Age at menarche (AAM) is related to many common female health problems. AAM is mainly determined by genetic factors. However, the specific genes and the associated mechanisms underlying AAM are largely unknown. Here, taking advantage of the most recent technological advances in the field of human genetics, we identified multiple genetic variants in a gene, *SPOCK*, which are associated with AAM variation in a group of Caucasian women. This association was subsequently confirmed not only in two independent groups of Caucasian women but also across ethnic boundaries in one group of Chinese women. In addition, *SPOCK* has a function in regulating a key factor involved in menstrual cycles, *MMP-2*, which provides further support to our findings. Our study provides a solid basis for further investigation of the gene, which may help to reveal the underlying mechanisms for the timing of menarche and for AAM's relationship with women's health in general.

## Introduction

Menarche is a most significant milestone in a female's physiological development. It happens when thickened endometrial tissue undergoes a sudden death due to fluctuations of hormone levels. Age at menarche (AAM) has a significant impact on a woman's health later in life. For example, early AAM is associated with breast and endometrial cancers [1],[2] and late AAM increases the risk of Alzheimer's disease [3] and osteoporosis [4],[5]. Overall, early AAM appear to be more harmful to women's health than late AAM. In a cohort containing >61,000 Norwegian women, it was found that there was an inverse association between AAM and an all-cause mortality rate, with each year decrease in AAM associated with an average increase of 2.4% in mortality rate [6]. The significant health implications of AAM make it an interesting and important trait to study. Understanding the determining factors of AAM may shed light on the etiology of AAM-associated diseases and women's health in general.

Genetic factors play a dominant role in determination of AAM. From 50% to 70% variation of AAM can be explained by genetic factors [7]–[9]. However, specific genes underlying AAM are largely unknown. So far, compared with other human complex diseases/traits, a limited few studies were performed to dissect the genetic basis of AAM. A few candidate genes were suggested to influence AAM, e.g., the estrogen receptor alpha (*ER-α*) and beta (*ER-β*) genes [10]–[12], the *SHBG* gene [13], the androgen receptor gene [14], the insulin-like growth factor 1 (*IGF-1*) gene [15], the chemokine (C-C-motif) receptor 3 (*CCR3*) gene [16], and the genes of CYP family [17]–[19]. To date, only three genome-wide linkage studies on AAM were published [9],[20],[21], including one by our own group [20]. The studies identified several genomic regions (e.g., 22q11, 22q13, 16q12, 16q21 and 12q) that may harbor QTLs (quantitative trait loci) underlying AAM.

A promising strategy to facilitate identification of AAM genes is a genome-wide association study (GWAS) that takes advantage of the knowledge of linkage equilibrium (LD) patterns in humans and the rapid development of high throughput SNP genotyping platforms. With high SNP density that makes possible the detection of culprit DNA changes within a narrow genomic region, the GWAS approach has demonstrated its great power to identify novel genes for human complex diseases/traits [22]–[26].

In this study, we conducted a GWAS to search for novel genetic factors underlying AAM. Using Affymetrix 500 K array, we successfully genotyped and analyzed a total of 379,319 SNPs in a cohort of 477 unrelated women, all ascertained with AAM data. We identified seven SNPs of a novel gene, *SPOCK* (Sparc/Osteonectin, CWCV, and Kazal-like domains proteoglycan), which were associated with AAM at genome-wide significance level. We selected six most significant SNPs of the gene and successfully replicated them within and/or across ethnic boundaries in three independent cohorts, including one Chinese cohort containing >1,300 subjects and two independent Caucasian cohorts with a total of 1,616 subjects. Our study provides strong evidence for the gene's importance in regulation of timing of menarche.

## Results

### Analysis for Population Stratification

To detect potential stratification of our GWAS Cohort, we analyzed the sample using software Structure 2.2 [27]. When 200 randomly selected un-linked markers were used to cluster our subjects, under all the assigned values (i.e., 2, 3, and 4) for the assumed number of population strata, *k*, all the subjects of the cohort were tightly clustered together, suggesting no population stratification. The results are shown in Figure S1.

We further tested our GWAS Cohort for population stratification using the genomic control method [28]. Based on genome-wide SNP information, we estimated the inflation factor (λ), a measure for population stratification. Ideally, for a homogeneous population with no stratification the value of λ should be equal or near to 1.0. In the GWAS Cohort, the estimated value for λ was 1.000, which suggested essentially no population stratification and further confirmed the results achieved through the Structure 2.2 software.

### Q/Q Plot Analysis

Using the Q-Q plot, we examined the distribution of the *p* values achieved in our GWAS for all the analyzed ∼380,000 SNPs (Figure S2). As shown in the plot, the observed *p* values match reasonably well with the expected *p* values over a wide range of values of [−LOG_10_(*p*)], which is from 0 to ∼4.5. Observed *p* values gradually depart from expected *p* values at the extreme tail, where [−LOG_10_(*p*)] is ≥∼4.5. The pattern suggests that our GWAS association findings were more likely due to true genetic variation than other reasons such as genotyping errors, sample relatedness or potential population stratifications.

### Association Analyses in GWAS Cohort

**Figure 1:**
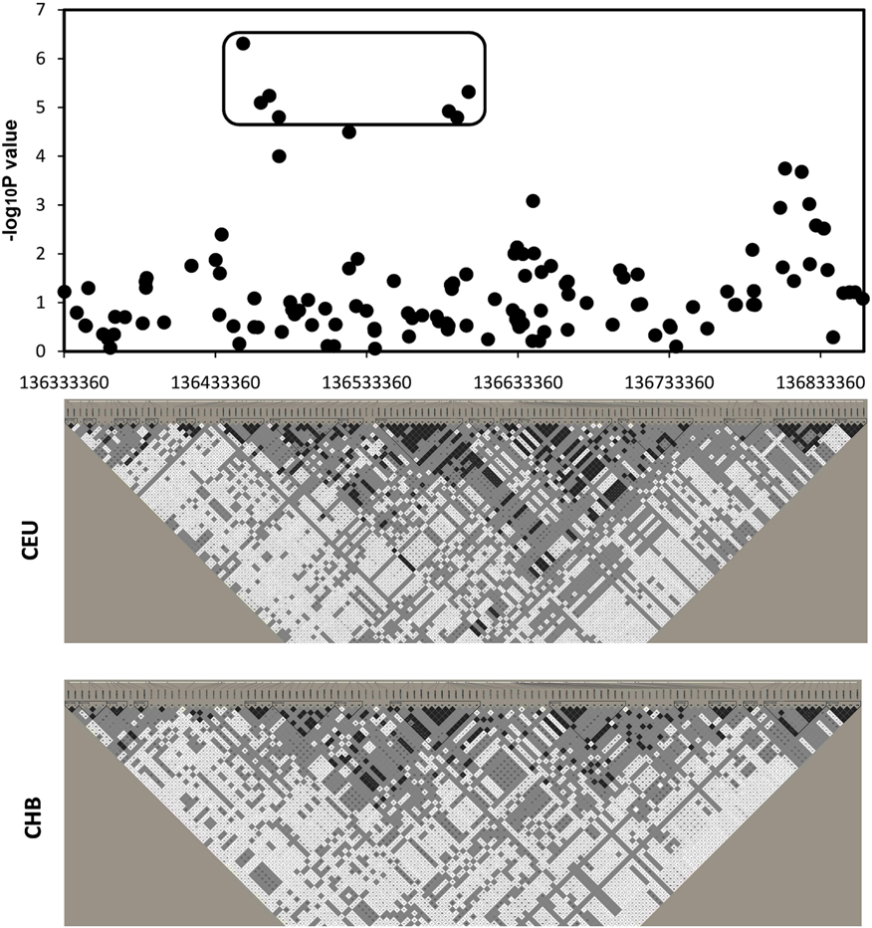
Association signals of the *SPOCK* gene SNPs. This figure depicts association signals achieved in Caucasians for all the genotyped SNPs of the *SPOCK* gene in our GWAS. The X axis shows the physical position of each SNP. The haplotype block map for the whole span of the *SPOCK* gene, showing pairwise LD in *D′*, was constructed for both Caucasians (CEU) and Chinese Han (CHB) using the Haploview program [54] (http://www.broad.mit.edu/mpg/haploview/) and the most recent SNP genotype data (HapMap Data Rel 26/phaseIII Nov 08, on NCBI B36 assembly, dbSNP b126) from HapMap (www.hapmap.org). The seven SNPs circled within the rounded rectangle are those achieving significant genome-wide FDR values (*q*<0.05), which are further illustrated in Figure 2.

**Figure 2:**
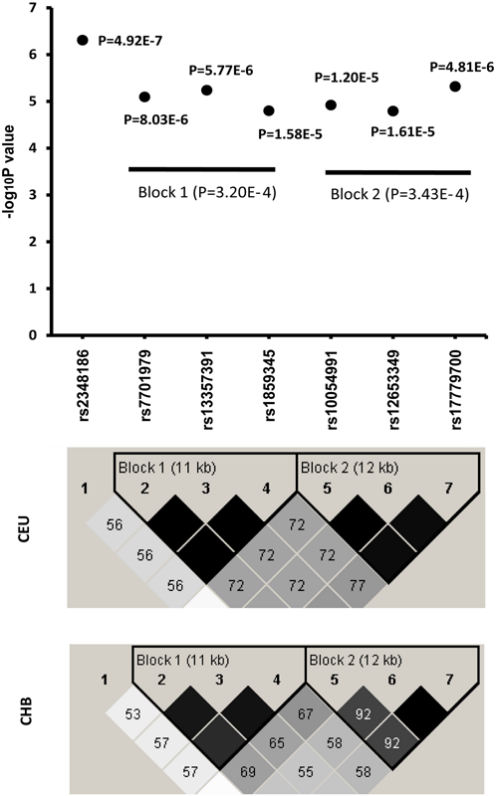
Association signals of the seven most significant SNPs of the *SPOCK* gene. This figure illustrates the association signals in our GWAS for the seven *SPOCK* gene SNPs that achieved significant genome-wide FDR values (*q*<0.05). The figure also shows the signals for two haplotype blocks (Blocks 1 & 2) formed by the SNPs *rs7701979*, *rs13357391* and *rs1859345* (for Block 1) and the SNPs *rs10054991*, *rs12653349* and *rs17779700* (for Block 2). The haplotype block map for the seven *SPOCK* gene SNPs, showing pairwise LD in *D′*, was constructed for both Caucasians (CEU) and Chinese Han (CHB) using the Haploview program [54] (http://www.broad.mit.edu/mpg/haploview/) and the most recent SNP genotype data (HapMap Data Rel 26/phaseIII Nov 08, on NCBI B36 assembly, dbSNP b126) from HapMap (www.hapmap.org).

We performed genome-wide genotypic association analyses for AAM in our GWAS cohort. The detailed characteristics for the study subjects are presented in Table 1. We used the FDR-based *q* value to control the genome-wide significance for the identified SNP markers at the significance level of *q* = 0.05. Using this threshold, we identified a total of 21 markers of known genes (Table S1) among a total of ∼380,000 tested markers. Of note, one third (i.e., seven) of these 21 markers belong to the *SPOCK* (Sparc/Osteonectin, CWCV, and Kazal-like domains proteoglycan) gene. We therefore focused our subsequent analyses on this gene. Table S2 lists the relevant information of all the 130 genotyped SNPs of this gene, including the raw *p* values achieved, with the most significant seven SNPs that passed the FDR *q* threshold of 0.05 highlighted in bold. For readers' convenience, we listed in Table 2 only these seven SNPs, with their detailed FDR *q* values. Figure 1 plots the 130 SNPs and Figure 2 the seven most significant SNPs of the gene, showing negative Log_10_P values achieved at these SNPs.

**Table 1:** Characteristics of the subjects for GWAS and the replication study.

Age Range	GWAS Cohort (Caucasian)		Replication Cohort I (Caucasian)		Replication Cohort II (Caucasian)		Replication Cohort III (Chinese)	
	N	AAM (yr)	N	AAM (yr)	N	AAM (yr)	N	AAM (yr)
<40	175	13.0 (1.5)	426	12.8 (1.4)	6	12.8 (2.2)	943	13.4 (1.4)
40–50	58	12.7 (1.4)	329	13.0 (1.6)	98	12.6 (1.4)	97	13.7 (1.7)
50–60	37	13.5 (4.6)	77	13.4 (1.6)	247	12.8 (1.5)	174	14.5 (1.8)
≥60	207	12.9 (1.4)	22	12.8 (1.5)	411	12.8 (1.5)	173	15.1 (1.9)
Total	477		854		762		1,387	

Note: Presented are means (SD).

**Table 2:** SNPs identified in GWAS with genome-wide significant FDR *q* values.

SNP Name	Position	Role	Allele^1^	MAF^2^	MAF^3^	*p* value	FDR *q* value	SNP effect size^4^			
								β	SE	R^2^	Reference allele
*rs2348186*	136451658	Intron 5	T/C	0.464	0.492	4.92×10^−7^	0.035	−0.821	0.158	0.059	C
*rs7701979*	136463382	Intron 5	G/T	0.367	0.308	8.03×10^−6^	0.038	−0.644	0.144	0.045	T
*rs13357391*	136468981	Intron 5	T/C	0.344	0.308	5.77×10^−6^	0.038	−0.644	0.144	0.044	C
*rs1859345*	136475319	Intron 5	T/C	0.343	0.308	1.58×10^−5^	0.044	−0.624	0.144	0.042	C
*rs10054991*	136587711	Intron 3	A/G	0.235	0.233	1.20×10^−5^	0.042	−0.605	0.143	0.040	G
*rs12653349*	136593147	Intron 3	A/G	0.237	0.233	1.61×10^−5^	0.044	−0.605	0.143	0.040	G
*rs17779700*	136600692	Intron 3	A/G	0.233	0.208	4.81×10^−6^	0.038	−0.604	0.143	0.040	G

1 The second allele represents the minor allele of each marker.

2 Minor allele frequency calculated in our cohort.

3 Minor allele frequency reported for Caucasians in the HapMap CEU.

4 The association analysis and estimation for SNP effect size was performed under dominant genetic model.

Among these seven SNPs of the *SPOCK* gene, four are located in intron 5 and three are located in intron 3 (Table 2). Haplotype analyses indicated that *rs2348186* stands alone with weak LD with the other six SNPs that form two haplotype blocks, with one (Block 1) containing three SNPs in intron 5, *rs7701979*, *rs13357391*, and *rs1859345*, and the other (Block 2) containing three SNPs in intron 3, *rs10054991*, *rs12653349*, and *rs1779700* (Figure 2). These two blocks have suggestive association with AAM, with Block 1 and Block 2 achieving *p* values of 3.20×10^−4^ and 3.43×10^−4^, respectively (Figure 2). The more detailed haplotype block structure of the seven SNPs showing both r^2^ and D′ matrixes is presented in Figure S3.

Using each SNP as a covariate, we performed conditional analysis on the remaining 6 SNPs for their association with AAM. This analysis is to investigate if there is one SNP among the 7 SNPs of the *SPOCK* gene, which can explain on its own the association signals of other 6 SNPs. To simplify the presentation without losing generality, we only present the results (in Table S3) for the analyses conditioning on each of the three SNPs, *rs2348186*, *rs13357391* and *rs12653349*, which are located at different haplotype blocks (as shown in Figure S3) and have relatively low LD between each other. Overall, there is significant drop of association signals in the conditional analysis as compared with the regular association analysis, suggesting that the association signals between these 7 SNPs are highly correlated. In addition, there is a clear pattern that the stronger the LD between a SNP and the SNP used as the covariate for conditional analysis, the larger drop of the association signals for the former SNP, suggesting the correlation of the association signals between these 7 SNPs is largely due to the LD between them. However, there is no SNP that when used as a covariate for conditional analysis, had made the association signals (achieved in regular association analysis) disappear for all the remaining 6 SNPs, suggesting that none of the 7 SNPs can explain on its own the association signals for all of the other 6 SNPs.

### Replication Analyses

Among these seven SNPs, we selected six most significant SNPs for replication, including four in intron 5 (*rs2348186*, *rs7701979*, *rs13357391*, and *rs1859345*) and two in intron 3 (*rs10054991* and *rs17779700*). The association results achieved in the three replication cohorts are summarized in Table 3. According to the results, all the six SNPs were replicated (achieving replication *p* values<0.05) in ≥ one replication cohort and four were replicated in ≥ two replication cohorts. In particular, three SNPs in intron 5 (*rs7701979*, *rs13357391* and *rs1859345*) that formed the Block 1 as determined in our GWAS (Figure 2) as well as the block itself were replicated in both of the two Caucasian replication cohorts (Replication Cohorts I & II) (Table 3). Interestingly, Four SNPs (*rs13357391*, *rs1859345*, *rs10054991*, and *rs17779700*), including two (*rs13357391* and *rs1859345*) in intron 5 and two (*rs10054991* and *rs17779700*) in intron 3, were replicated across ethnic boundaries in Chinese (Replication Cohort III) (Table 3). Overall, the strongest replication signals were achieved for the two SNPs in intron 5, *rs13357391* and *rs1859345*, which were replicated both within and across ethnic groups in all three replication cohorts, achieving *p* values of 5.09×10^−3^ and 4.37×10^−3^, respectively, in the Chinese replication cohort and combined *p* values of 5.19×10^−5^ and 1.02×10^−4^, respectively, in the analyses of all three replication cohorts (Table 3).

**Table 3:** Association signals of SNPs under replication.

SNP name	Allele	Replication Cohort I (Caucasian)		Replication Cohort II (Caucasian)		Replication Cohort III (Chinese)		Combined *p* values in 2 Caucasian replication cohorts		Combined *p* values in all 3 replication cohorts
		MAF	*p* value	MAF	*p* value	MAF	*p* value	Fisher's	UNPHASED	
*rs2348186*	T/C	0.481	0.15	0.479	**0.048**	–	**–**	**0.043**	**9.12×10^−3^**	**–**
*rs7701979	G/T	0.297	**0.024**	0.297	**0.049**	–	**–**	**9.11×10^−3^**	0.24	**–**
*rs13357391	T/C	0.303	**0.011**	0.305	**7.50×10^−3^**	0.160	**5.09×10^−3^**	**8.58×10^−4^**	**3.75×10^−4^**	**5.19×10^−5^**
*rs1859345	T/C	0.307	**0.015**	0.305	**0.014**	0.163	**4.37×10^−3^**	**1.99×10^−3^**	**9.16×10^−4^**	**1.02×10^−4^**
*rs10054991*	A/G	0.202	0.27	0.200	0.057	0.123	**0.028**	0.080	**0.035**	**0.017**
*rs17779700*	A/G	0.203	0.33	0.198	**0.045**	0.138	**0.022**	0.077	0.053	**0.013**
*Block 1*	**–**	**–**	**0.014**	**–**	**0.021**	–	**–**	**–**	**–**	**–**

Block 1 is formed by the three neighboring SNPs in intron 5 marked with asterisks. The second allele represents the minor allele of each marker. MAF, minor allele frequency calculated based on the genotypes of our study subjects. Marked in bold are the replication *p* values less than 0.05. Combined *p* values in 2 Caucasian replication cohorts were calculated using Fisher's method [29] or using the UNPHASED software (through association analysis on pooled sample containing the two cohorts) [58]. Combined *p* values in all 3 replication cohorts were calculated using Fisher's method only. The two SNPs, *rs2348186* and *rs7701979*, deviate from HWE (*p*<0.001) in the Chinese cohort and hence were excluded from association analyses. The association analysis for Replication Cohorts I and II was performed under the dominant model. The analysis for Replication Cohort III was performed under the recessive model.

Using the linear regression function in SAS (SAS Institute Inc., Cary, NC), we estimated the effect sizes of the significant SNPs detected/replicated in our GWAS and the two replication cohorts containing random unrelated subjects (Replication Cohorts II and III). The results of the estimation are presented in Tables 2 and 4. Since currently there is no convenient method and software to estimate the effect size for a SNP in a family-based sample, we did not perform effect size estimation for Replication Cohort I that is made up of siblings. Based on the analysis, the effect for each of the SNPs under replication is in the same direction in GWAS as in our replication cohorts; carriers of the minor allele tend to have a lower AAM than non-carriers in all the cohorts.

**Table 4:** Magnitude and direction of SNP effects in Replication Cohorts II and III.

SNP name	Replication Cohort II (Caucasian)			Replication Cohort III (Chinese)			Reference allele
	β	SE	R^2^	β	SE	R^2^	
*rs2348186*	−0.215	0.140	0.005	–	–	–	C
*rs7701979*	−0.159	0.125	0.003	–	–	–	T
*rs13357391*	−0.267	0.123	0.009	−0.709	0.271	0.005	C
*rs1859345*	−0.245	0.124	0.008	−0.791	0.262	0.007	C
*rs10054991*	−0.237	0.129	0.006	−0.784	0.393	0.003	G
*rs17779700*	−0.232	0.128	0.006	−0.694	0.333	0.003	G

The two SNPs, *rs2348186* and *rs7701979*, deviate from HWE (*p*<0.001) in the Chinese cohort and hence were excluded from association analyses. The estimation for SNP effect size for Replication Cohort II was performed under the dominant model. The analysis for Replication Cohort III was performed under the recessive model.

We examined AAM association for the haplotype formed by the two haplotype blocks, Block 1 (containing *rs7701979*, *rs13357391*, and *rs1859345*) and Block 2 (containing *rs10054991*, *rs12653349*, and *rs17779700*), as shown in Figure 2, in our GWAS as well as three replication cohorts. The results are shown in Table 5. According to the analysis, two haplotypes formed by the two blocks achieved *p* values of ∼8.0×10^−4^ in the GWAS Cohort. In the replication cohorts, the signals were most significant in Replication Cohort I, where two haplotypes both achieved a *p* value of <0.05. Overall, association signals achieved through the haplotype analysis were less significant than through single SNP analysis.

**Table 5:** Association analysis of haplotypes formed by SNPs of the *SPOCK* gene.

Cohort	Component SNPs of a Haplotype	Haplotype	Haplotype Frequency	*p* value for AAM association
GWAS Cohort	SNPs 1-2-3-4-5-6	G-A-T-T-T-A	0.605	7.90×10^−4^
		T-G-C-C-C-G	0.197	8.04×10^−4^
Replication Cohort I	SNPs 1-2-3-4-6	G-C-T-A-G	0.677	0.038
		T-C-C-A-A	0.123	0.015
Replication Cohort II	SNPs 1-2-3-4-6	G-A-T-A-T	0.659	0.069
		T-G-C-G-C	0.166	0.093
Replication Cohort III	SNPs 2-3-4-6	A-A-C-C	0.066	0.051
		A-A-T-T	0.786	0.054

SNP1: *rs7701979*, SNP2: *rs13357391*; SNP3: *rs1859345*; SNP4: *rs10054991*; SNP5: *rs12653349*; SNP6: *rs17779700*. The haplotype covers the two haplotype blocks, Block 1 and Block 2, as shown in Figure 1. SNP 5 was not genotyped in Replication Cohorts I and II, and SNPs 1 and 5 were not genotyped in Replication Cohort III. Therefore, these SNPs were not included as the component SNPs for the haplotype analysis for the Replication Cohorts.

**Figure 3:**
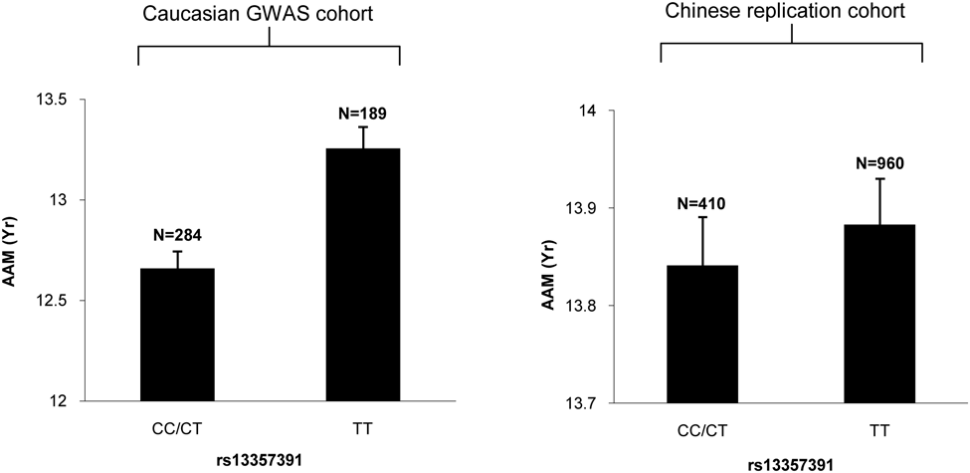
Comparison of AAM among subjects of different genotypes at *rs13357391*. Presented in the figure are the means and standard errors of AAM for specific genotype groups in the GWAS and Chinese replication cohorts.

Figure 3 presents the AAM data among both Caucasian and Chinese subjects of different genotypes at the SNP *rs13357391* that achieved the most significant combined *p* value in the entire replication analyses. As shown here, although Chinese subjects have higher average AAM than Caucasians, the AAM difference between the two genotype groups {C/C(T) vs. T/T} persists across the two ethnic groups.

### Other Analyses

Using Genetic Power Calculator (http://pngu.mgh.harvard.edu/˜purcell/gpc/), we calculated the power of the sample used in the GWAS. Assuming there are 10 QTLs (quantitative trait loci) each with an allele frequency of 0.2 and in complete LD with the SNPs under examination, and each contributing 1.5% variance of AAM, under the significance level of 5×10^−7^, our sample will have >70% statistical power to identify at least one such QTL. If under the significance level of 1×10^−6^, then our sample will have >80% statistical power to identify at least one such QTL.

Using FASTSNP program, we investigated the potential functions of the replicated SNPs of the *SPOCK* gene. According to the analyses, three replicated SNPs (*rs7701979*, *rs1859345* and *rs17779700*) are located at intronic enhancer regions and may cause change in transcription factor binding efficiency; a “G→T” change at *rs7701979* may lead to removal of the binding site for the transcription factor *v-Myb*, whereas a “T→C” change at *rs1859345* and an “A→G” change at *rs17779700* may lead to creation of the binding site for the transcription factor *GATA-1*.

To evaluate in our GWAS dataset previously identified candidate genes for AAM (including the *ER-α* and *ER-β* genes [10]–[12], the *SHBG* gene [13], the androgen receptor gene [14], the *IGF-1* gene [15], the *CCR3* gene [16], and the genes of CYP family [17]–[19]), we checked the *p* values of the SNPs in or near those genes for association with AAM in our GWAS cohort. We found that three SNPs in or near the *CCR3* gene achieved nominally significant *p* values; a SNP *rs13084656*, located in the promoter region, achieved a *p* value of 0.039; another SNP *rs2157056*, located downstream of the gene, achieved a *p* value of 6.27×10^−3^; a third SNP *rs2373156*, located in the intronic region, achieved a *p* value of 0.050. For other genes listed above, we did not find SNPs with positive association signals (*p*<0.05) in our GWAS dataset.

The above three *CCR3* gene SNPs were not genotyped in the previous *CCR3* candidate gene study for AAM [16], where only 16 other SNPs of the gene were genotyped. To facilitate comparison between this GWAS and the previous study [16] in terms of *CCR3* gene's association with AAM, we performed an imputation-based meta-analysis. Based on the genotype data generated in our GWAS for those SNPs in or surrounding the *CCR3* gene, we imputed a total of 1,380 SNPs to cover the gene and its neighborhood regions, among which, 9 imputed SNPs were also genotyped in the previous *CCR3* candidate gene study [16]. The association signals achieved at these 9 imputed SNPs in our GWAS sample were compared with the signals achieved in the previous candidate gene study [16] in Table 6. As shown in the table, the reliability of the imputed genotypes at these 9 SNPs is evidenced by the similarity between the MAFs (minor allele frequency) derived from the imputed genotype data in the GWAS sample and the MAFs derived from the genotype data generated experimentally in the previous *CCR3* candidate gene study [16]. Several imputed SNPs, i.e., *rs4987053* in exon 3, *rs17217831* in intron 2, *rs13067058* in promoter region, and *rs3091309* in intron 1, achieved significant (*p*<0.05) or marginally significant (*p*<0.10) *p* values in our GWAS sample. In particular, the SNP, *rs3091309*, achieved an imputed *p* value of 0.094 in our GWAS sample and a *p* value of 0.006 in the previous study [16]. The direction of the SNP effects is also the same in the two studies, where carriers of the minor allele “A” tend to have a lower AAM as compared with the non-carriers. Using Fisher's method [29] to combine the two *p* values achieved, a *p* value of 4.78×10^−3^ was obtained for the overall association of this SNP with AAM in the two studies. Our meta-analysis results provide further support for the *CCR3* gene's importance to AAM as suggested in the previous study [16].

**Table 6:** Comparison between previous study and this GWAS for *CCR3* gene's association with AAM.

SNP name	Results in the previous study [16]						Imputed results in GWAS sample	
	Position	Role	Allele^1^	MAF^2^	MAF^3^	*p* value	MAF^4^	*p* value
*rs13067058*	46248770	promoter	G/A	0.074	0.067	0.142	0.076	0.066
*rs1388604*	46251882	promoter	T/A	0.365	0.325	0.748	0.345	0.921
*rs6441948*	46270781	intron 1	G/A	0.443	0.479	0.009	0.446	0.200
***rs3091309*** 5	46278188	intron 1	G/A	0.191	0.208	**0.006**	0.200	**0.094**
*rs1491962*	46279610	intron 2	T/C	0.445	0.460	0.03	0.448	0.277
*rs17217831*	46280445	intron 2	C/A	0.073	0.067	0.151	0.076	0.050
*rs4987053*	46281704	exon 3	T/C	0.075	0.067	0.663	0.077	0.031
*rs3091312*	46283476	downstream	T/A	0.249	0.250	0.503	0.256	0.858
*rs1027241*	46287543	downstream	G/A	0.446	0.400	0.06	0.450	0.301

1 The second allele represents the minor allele of each marker.

2 Minor allele frequency calculated in the sample of the previous study [16].

3 Minor allele frequency reported for Caucasians in the HapMap CEU.

4 Minor allele frequency calculated based on imputed genotypes.

5 The direction of the effects for the SNP, *rs3091309*, is the same in the previous study as in the GWAS sample according to the imputation results, where carriers of the minor allele “A” tend to have a lower AAM as compared with the non-carriers. Using Fisher's method [29] to combine the two *p* values achieved in the previous and the current GWAS samples, a *p* value of 4.78×10^−3^ is obtained for the overall association of this SNP with AAM in the two datasets.

## Discussion

This study reports the first GWAS of AAM in a Caucasian cohort. Through this study, we identified a novel gene, *SPOCK*, with multiple SNPs associated with AAM variation at the genome-wide significance level (FDR *q* value<0.05). Noticeably, a group of SNPs of the *SPOCK* gene consistently showed genome-wide significance at the threshold of genome-wide FDR *q*<0.05 (Table S1). Our GWAS and the follow-up independent replication study corroborated the *SPOCK* gene's importance for AAM variation in both Caucasians and Chinese.

*SPOCK* is a proteoglycan isolated first in human testes and hence initially named “testican” [30]. So far, its functions are still largely unknown. Highly expressed in the brain [31], *SPOCK* was also found in other tissues, including cartilage [32], vascular endothelium [33], myoblasts [34], fibroblasts [35], lymphocytes [36], and neuromuscular junction [37]. Recently, *SPOCK* was also identified in the blood [38], suggesting that it may exert its functions at the systemic level. The most noticeable finding for this gene is its inhibition of *MMP-2* (matrix metalloproteinase-2) activation [39]. Interestingly, *MMP-2* was found to be a key factor mediating cyclic endometrial menstrual breakdown and onset of menstrual bleeding [40]. Taking into account our association findings, *SPOCK* may play an essential role in AAM regulation through its inhibition of *MMP-2*. However, the above mechanism is still speculative and needs extensive functional studies for final validation.

This study also provided some support for the previous findings on the *CCR3* gene's importance to AAM [16]. Through imputation-based association analysis, we identified marginally significant association signals (*p* = 0.094) in the GWAS sample for a SNP, *rs3091309*, that was also associated with AAM in the previous *CCR3* candidate gene study [16], achieving a *p* value of 0.006. The SNP's direction of effects for AAM association is also the same between this GWAS and the previous study [16]. The combined *p* value at this SNP for the overall association with AAM in the two studies reaches 4.78×10^−3^.

Imperfect data collection exists more or less in studies of almost all human diseases/traits. In our study, the AAM data were collected through retrospective self-reporting. Statistically, there is no systematic bias in reporting AAM in studies of this trait. Therefore, inaccuracy of the AAM recall data may be factored into random noise in the data collected and may only have a potential effect to decrease the power for detecting the AAM genes. However, the inaccuracy, if not systematically biased, should not render false positive findings.

The overall precision of our AAM data is partially supported by a high heritability of ∼0.60 for AAM as detected in our previous WGLS of AAM [20], where we used the same approach in AAM data collection as in the current study. Importantly, such a high heritability could not be achieved if significant errors and thus noise existed in the AAM data of our study subjects. The reliability of our AAM data is further supported by our replication results. As shown in Table 1, our GWAS findings were achieved in a cohort mostly composed of aged subjects (with ages >50 years). However, we replicated our most significant GWAS findings using cohorts containing mainly younger subjects. Our largest replication cohort, the Chinese cohort, has ∼70% subjects younger than 40. Our second largest replication cohort, the Replication Cohort I, has ∼50% subjects younger than 40 and ∼90% subjects younger than 50. The success in replicating findings mainly from older subjects using mostly younger subjects implicates the high reliability of AAM recollection for our study subjects.

Our GWAS sample was initially designed for studying not only AAM but also other complex diseases/traits, such as osteoporosis, obesity, and human height. The original sample contained 1,000 subjects, with ∼500 females and ∼500 males, who have multiple phenotypes such as bone mineral density, body mass index, fat mass, lean mass, and height, etc [41],[42]. Among the subjects, only 477 females have well-substantiated AAM phenotype and can be used for a GWAS study of AAM. Therefore, our GWAS cohort contains only 477 subjects. Due to polygenic nature of AAM and therefore, the existence of multiple QTLs for the trait, even with such a small size, our GWAS sample may still have a reasonable power to detect at least one of the QTLs (see the detailed power calculation in the Results Section). This is evidenced by our success in identifying the *SPOCK* gene using the sample. Moreover, the robustness of our finding on the gene is testified by its replication in several other cohorts.

Nevertheless, the small size of the GWAS cohort is still an limitation of our study, which may have limited the statistical power to identify more potentially important genes underlying AAM. In addition, the detailed mechanism of the identified gene, *SPOCK*, on AAM regulation is still unclear and therefore, the direct functional relevance of the gene to AAM cannot be fully determined. To address the above limitations, our future research will be directed to the following aspects. First, a new GWAS needs to be implemented on a larger sample to identify more comprehensively novel genes for AAM. Second, data from this study will be shared with other groups in the AAM research community to build a larger dataset for a GWAS-based meta-analysis. Lastly, further functional and molecular studies will be performed on the *SPOCK* gene to analyze and reveal the specific mechanisms of the gene in regulating timing of menarche.

In summary, we identified a novel gene, *SPOCK*, for AAM through a GWAS and replicated both within and across ethnicity its association with AAM in independent cohorts. Functional relevance of *SPOCK* to AAM is supported by its well documented role in inhibition of a menstrual proteinase, *MMP-2*. Our finding furnishes a solid basis for further molecular and functional analyses of the gene to pursue its more detailed functions in regulating timing of menarche and women's health in general.

## Materials and Methods

### Study Populations

The study was approved by the Institutional Review Board and/or the Department of Research Administration of the involved institutions. Signed informed-consent documents were obtained from all study participants before they entered the study. A cohort containing 477 unrelated women (i.e., GWAS Cohort) was selected for our GWAS from our established and expanding genetic repertoire currently containing more than 6,000 subjects recruited in Midwestern USA in Omaha, NE and its surrounding areas. For replication of our GWAS findings, we selected two independent cohorts from the same genetic repertoire, one containing 854 siblings (Replication Cohort I) and another containing 762 unrelated women (Replication Cohort II). The inclusion criteria for the above cohorts include: 1) Caucasians of European origin; 2) healthy female subjects with regular menses or if postmenopausal, with a history of regular menses throughout the years before menopause; and 3) without diseases and conditions that may potentially affect regular menstrual cycles, as listed in the exclusion criteria. The detailed exclusion criteria were published elsewhere [43],[44]. Briefly, subjects with chronic diseases and conditions involving vital organs (heart, lung, liver, kidney, and brain) and severe endocrinological, metabolic, and nutritional diseases that might affect regular menstrual cycles were excluded from this study. The general relevant characteristics of the study subjects are listed in Table 1. The above three cohorts were selected based on the criterion that the subjects in different cohorts should not overlap or be related. Therefore, these three cohorts, although selected from the same large genetic repertoire containing 6,000 subjects, are independent and unrelated.

Another cohort from China (Replication Cohort III) was also used to replicate our GWAS findings and to assess ethnic specificity/generality of the findings in Caucasians. The cohort contains unrelated 1,387 female subjects of Chinese Han ethnicity recruited from Cities of Changsha and Xi'an and their surrounding areas. Except for the requirement for recruiting subjects of Chinese Han ethnicity, the inclusion and exclusion criteria for Replication Cohort III were the same as for our Caucasian subjects. The general relevant characteristics of these Chinese study subjects are also listed in Table 1.

AAM data of all the female subjects in the above four cohorts (i.e., the GWAS Cohort and Replication Cohorts I, II & III) were collected based on a same standard nurse-administered questionnaire, which included a detailed medical and female history. In particular, the questionnaire administered to the Chinese subjects was the accurate Chinese translation of the original questionnaire used by the Caucasian subjects. All the study subjects reported their AAM to the accuracy of one year. (Although the raw AAM data are integers, in Table 1 and Figure 3, the AAM values presented are not, which is caused by averaging the raw AAM data among subjects in a certain group.)

Unlike for other traits, recollection by subjects is a generally reliable measure for AAM data collection. This is because AAM is a most significant event in female puberty, which often has a major impact on a woman's life, both physically and psychologically. A recent study found a high correlation of ∼0.80 between the original AAM and the AAM recalled even 30 years later [45]. Consistent with the finding, several other studies also indicated reliability of the retrospective method in AAM data acquisition [46]–[48]. Therefore, our study followed this common practice in the field, which is feasible, convenient and accurate to perform.

### Genotyping

#### Genotyping for the GWAS Cohort

Genomic DNA was extracted from whole human blood using a commercial isolation kit (Gentra systems, Minneapolis, MN, USA) following the protocols detailed in the kit. Genotyping with the Affymetrix Mapping 250 k Nsp and Affymetrix Mapping 250 k Sty arrays was performed using the standard protocol recommended by the manufacturer. Genotyping calls were determined from the fluorescent intensities using the DM algorithm with a 0.33 *P*-value setting [49] as well as the B-RLMM algorithm [50]. DM calls were used for quality control while the B-RLMM calls were used for all subsequent data analysis. B-RLMM clustering was performed with 94 samples per cluster.

In our GWAS genotyping experiment, following an Affymetrix guideline, we set a standard for the minimum DM call rate at 93% (or the maximum genotype missing rate at 7%) for a sample, considering all the SNPs in the two arrays, the 250 k Nsp and 250 k Sty arrays. More than 98% of all the subjects (i.e., 470 subjects among a total of 477 subjects) met this call rate standard. The remaining 7 samples that did not meet this standard however had one hybridized array passing or approaching this call rate standard (i.e., 93% of all the SNPs in the array were successfully called). Hence the genotype data in the array (with the higher call rate) for these 7 samples were also kept in the dataset for GWAS analysis. For all the 477 subjects, the average DM call rate reached >95%.

The final average BRLMM call rate across the entire cohort reached a high level of 99.14%. However, out of the initial full-set of 500,568 SNPs, we discarded 32,961 SNPs with sample call rate <95%, another 36,965 SNPs with allele frequencies deviating from Hardy-Weinberg equilibrium (HWE) (*P*<0.001) and 51,323 SNPs with minor allele frequencies (MAF) <1%. Therefore, the final SNP set maintained in the subsequent analyses contained 379,319 SNPs, yielding an average marker spacing of ∼7.9 kb throughout the human genome.

#### Genotyping for Caucasian replication cohorts (Replication Cohorts I & II)

Among the seven SNPs of the *SPOCK* gene that were found to be associated with AAM in our GWAS analyses, we genotyped six most significant SNPs in our two Caucasian replication cohorts (Replication Cohorts I & II). Genotyping was performed by KBioscience (Herts, UK) using a modified TaqMan-based assay. The detailed description of the genotyping method can be found at the company's website (http://kbioscience.co.uk/). An average genotyping call rate of 97.8% was achieved. In addition, the genotyping duplicate concordance rate was 99.7%. All the six SNPs genotyped were in HWE (*p*>0.05).

#### Genotyping for Chinese replication cohort

The same set of SNPs genotyped in the Caucasian replication cohorts was also genotyped in our Chinese replication cohort (Replication Cohort III). Genotyping was performed using a primer extension method with MALDI-TOF mass spectrometry on a MassARRAY system (Sequenom, Inc., San Diego, CA). An average genotyping call rate of 98.7% was achieved and the genotyping duplicate concordance rate was 99.2%. Two of the six genotyped SNPs (*rs2348186* and *rs7701979*) were not in HWE (*p*<0.001) and therefore were excluded from further association analyses.

### Statistical Analyses

#### Analysis for potential population stratification

To detect population stratification that could lead to spurious association results in GWAS analyses, Structure 2.2 (http://pritch.bsd.uchicago.edu/software.html) was used to investigate the potential substructure of our GWAS Cohort. The program uses a Markov chain Monte Carlo (MCMC) algorithm to cluster individuals into different cryptic sub-populations on the basis of multi-locus genotype data [51]. To ensure robustness of our results, we performed independent analyses under three assumed numbers of population strata, *k* (i.e., 2, 3, and 4), respectively, using 200 un-linked markers randomly selected genome-wide.

To confirm the results achieved through Structure 2.2, we further tested population stratification of our GWAS Cohort using a method of genomic control [28].

#### Q-Q plot analysis

We examined the distribution of the *p* values for all the analyzed ∼380,000 SNPs in our cohort using the quantile-quantile (Q-Q) plot. The Q-Q plot is for assessing the magnitude of and validating observed associations, compared with the expectations under no association. The plot is constructed with the observed association statistics (e.g., the *t* statistic) or −log_10_P values ranked in order from smallest to largest on the Y-axis and plotted against the null distribution (expected under the null hypothesis of no association) on the X-axis. A deviation from the identity line at lower expected values of −log_10_P or a test statistic may be due to reasons other than true associations, such as genotyping errors, sample relatedness or potential population stratifications.

#### Adjustment for secular trend

We did not detect a secular trend of AAM in our Caucasian cohorts (including the GWAS Cohort and Replication Cohorts I & II). However, a significant secular trend was detected in our Chinese replication cohort (i.e., Replication Cohort III). As shown in Table 1, on average, older Chinese subjects have higher AAM than their younger counterparts. To correct for this secular trend, we used MiniTab (Minitab Inc., State College, PA) software's linear regression function and adjusted the AAM raw data using age as a covariate for the Chinese subjects before association analyses. After such adjustment, older Chinese subjects will have lower AAM as compared to their raw AAM data, which may remove the confounding effects of the secular trend for AAM.

#### GWAS association analysis

Genome-wide association analyses, including genotypic association analyses and haplotype association analyses, were performed using HelixTree 5.3.1 (Golden Helix, Bozeman, MT). For the genotypic association analyses, linear regression was used as the basic statistical framework, where genotype was treated as the independent variable and AAM as the dependent variable, and AAM was modeled as a linear function of alternative genotypes at a certain SNP. For the haplotype association analyses, “sliding window” haplotype association test was implemented under the framework of the haplotype trend regression (HTR) approach [52]. Within the framework, along each chromosome a certain number of consecutive SNPs (a “sliding window”) can be selected to infer the haplotype probability for each individual based on the composite haplotype method [53]. Then, an HTR model was constructed and used to evaluate the association of AAM with a haplotype encompassing the selected SNPs.

The LD patterns of the interested genes were analyzed and plotted using the Haploview program [54] (http://www.broad.mit.edu/mpg/haploview/) and the most recent SNP genotype data (HapMap Data Rel 23a/phaseII Mar 08, on NCBI B36 assembly, dbSNP b126) from HapMap (www.hapmap.org).

We adopted a method proposed by Storey and Tibshirani [55] and used the related software QVALUE (http://genomine.org/qvalue/) to calculate an FDR (false discovery rate)-based *q* value for each tested SNP to evaluate the statistical significance at the genome-wide level for the GWAS results.

We investigated if there is one SNP among the 7 identified SNPs of the *SPOCK* gene, which can explain on its own the association signals of other 6 SNPs. Using each of the SNPs as a covariate, we performed conditional analysis on the remaining 6 SNPs for their association with AAM. The analysis was implemented with the software MiniTab (Minitab Inc., State College, PA).

#### Replication analysis

Association analyses for SNP replication data were performed using HelixTree 5.3.1 (Golden Helix, Bozeman, MT) on the three independent replication cohorts. For Replication Cohorts II and III, genetic association analysis was performed in the same fashion as for the GWAS cohort, where linear regression analysis was performed modeling genotype as the independent variable and AAM as the dependent variable. For the Replication Cohort I, a family-based cohort, genetic association analysis was performed using the PBAT package of the HelixTree, which analyzes association signals by correlating transmission of parental genotype to offspring with AAM.

Using HBAT in FBAT (ver. 2.02) (http://biosun1.harvard.edu/˜fbat/fbat.htm) [56] and PLINK (ver. 1.03) (http://pngu.mgh.harvard.edu/purcell/plink/) [57], we performed haplotype-based association analysis for Replication Cohort I and Replication Cohorts II and III, respectively.

To quantify the overall evidence of association in the entire replication analyses, Fisher's method [29] was used to combine the individual *p* values achieved in each of the replication cohorts. The method, also known as Fisher's combined probability test, is a meta-analysis technique for combining the results from independent statistical tests that have the same overall null hypothesis (*H_0_*) [29]. The method combines *p* values from different studies into one test statistic that has a chi-square distribution using the formula 
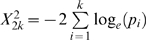
. The *p* value for the *X* ^2^ statistic can be extrapolated from a chi-square table using 2*k* “degree of freedom”, where *k* is the number of tests being combined.

To explore another method for combining the individual *p* values achieved in the two Caucasian replication cohorts (Replication Cohorts I and II), we pooled the two cohorts together and analyzed the pooled sample for association with AAM using the software, UNPHASED that allows for genetic association analysis of a sample containing both un-related (Replication Cohort II) and related subjects (Replication Cohort I) [58].

Using the linear regression model function in SAS (SAS Institute Inc., Cary, NC), we estimated the effect sizes of the significant SNPs detected/replicated in our GWAS and the two replication cohorts containing random unrelated subjects (Replication Cohorts II and III).

#### SNP functional analysis

To explore potential functions of the significant SNPs identified, we used FASTSNP (function analysis and selection tool for single nucleotide polymorphisms) (http://fastsnp.ibms.sinica.edu.tw) [59] that is a web server-based program designed for functional analysis of a SNP based on information extracted from various biological databases and analytical tools for SNP functional annotation. The program predicts the most likely functions of a SNP according to 13 putative functional effects, such as changes to the transcriptional level, pre-mRNA splicing, and protein structure alteration. The advantage of the program is that the functional prediction for a SNP is always based on the most up-to-date information extracted from 11 external web servers at the time of query. However, since the functional analysis of a SNP using FASTSNP is performed *in silico* based on the flanking sequence of the SNP, the predicted functions can only be viewed as putative and hypothetical and may need further support from experimental work.

#### Imputation analysis for the CCR3 gene

The three *CCR3* gene SNPs (*rs13084656*, *rs2157056*, and *rs2373156*) with positive association signals in our GWAS sample were not genotyped in the previous *CCR3* candidate gene study [16] for AAM. Therefore, to facilitate comparison between this GWAS and the previous study [16] in terms of the CCR3 gene's importance to AAM, we performed imputation analysis of the gene based on the genotype data generated in our GWAS. Using the software IMPUTE [60] (http://www.stats.ox.ac.uk/˜marchini/software/gwas/impute.html), we imputed a total of 1,380 SNPs covering the *CCR3* gene and its neighborhood regions. To ensure the reliability of the imputation, all of these imputed SNP markers have reached a calling threshold of 0.90, i.e., a 90% probability that an imputed genotype is true. Based on the imputed genotypes of these SNPs, we performed SNP association analyses using the software SNPTEST (http://www.stats.ox.ac.uk/˜marchini/software/gwas/snptest.html) to obtain the “imputed” *p* values for association with AAM at these SNPs in our GWAS sample.

## Supporting Information

Figure S1 Results of analyses of potential population stratification for the GWAS Cohort using Structure 2.2. As shown is output of the software Structure 2.2, which clustered our study subjects using 200 randomly selected unlinked markers under three assumed numbers of population strata, k = 2, 3, 4.
(0.06 MB PDF)

Figure S2 Q-Q plot for the *p* values achieved in the GWAS.
(0.03 MB PDF)

Figure S3 Haplotype block structure of the 7 AAM-associated *SPOCK* gene SNPs in Caucasians. The haplotype block structure was constructed using the HaploView software (http://www.broad.mit.edu/mpg/haploview/) [53] and the most recent SNP genotype data (HapMap Data Rel 26/phase III Nov 08, on NCBI B36 assembly, dbSNP b126) from HapMap (www.hapmap.org). The LD value between a certain pair of SNPs is shown within a corresponding “square”. A solid square without any value inside means a complete LD between the corresponding pair of SNPs.
(0.03 MB PDF)

Table S1 SNPs of known genes that passed genome-wide significant FDR threshold (*q*<0.05) in the GWAS.
(0.01 MB PDF)

Table S2 *SPOCK* SNPs and the *p* values for their association with AAM. Labeled in bold are SNPs that are significant at the genome-wide FDR level of 0.05 (q<0.05). ^1^The second allele represents the minor allele of each locus. ^2^Minor allele frequency calculated in our Caucasian study subjects. ^3^Minor allele frequency reported for Caucasians in the public database of HapMap CEU.
(0.03 MB PDF)

Table S3 Conditional association analysis of the 7 AAM-associated *SPOCK* gene SNPs in the GWAS cohort. Block 1 and block 2 are the haplotype blocks as shown in Figure S3. Original *p* values are the *p* values achieved in regular association analysis for AAM. *P* values for some SNPs cannot be estimated due to the high LD between the SNPs and the SNP used as the covariate for conditional analysis.
(0.02 MB PDF)
