# Supplementary figures and images for: Genome-Wide Association Analyses Identify SPOCK as a Key Novel Gene Underlying Age at Menarche
Source: PLoS Genet. 2009 Mar 13;5(3):e1000420. doi: 10.1371/journal.pgen.1000420 (PMC2652107; doi:10.1371/journal.pgen.1000420)

**Figure S1**

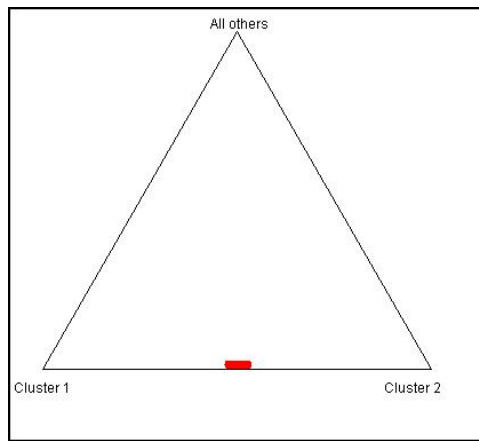

**k = 2**

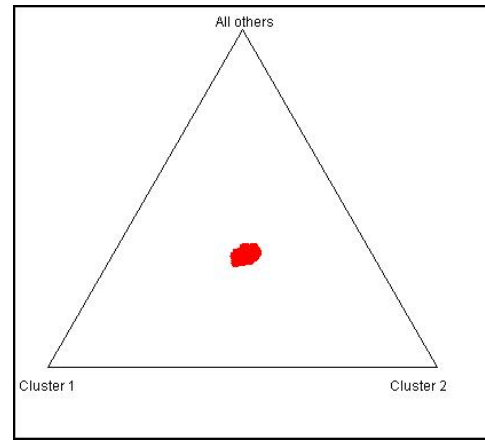

**k = 3**

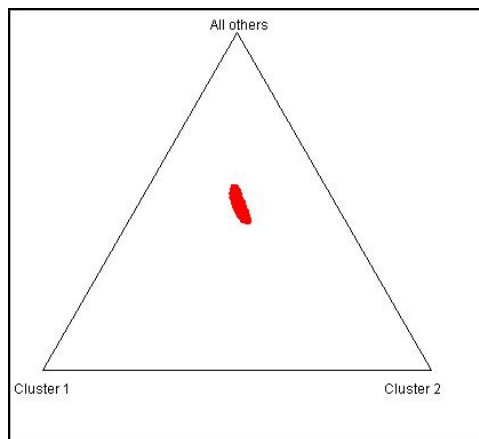

**k = 4**

Supplement: Figure S1 — Results of analyses of potential population stratification for the GWAS Cohort using Structure 2.2. As shown is output of the software Structure 2.2, which clustered our study subjects using 200 randomly selected unlinked markers under three assumed numbers of population strata, k = 2, 3, 4. (0.06 MB PDF) [file pgen.1000420.s001.pdf]

**Figure S2**

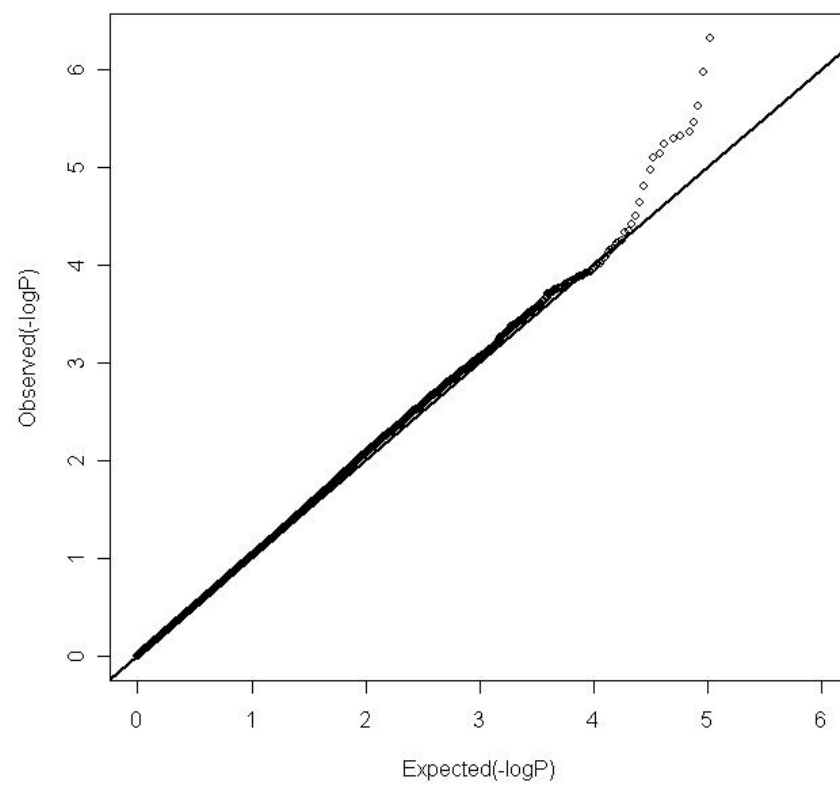

Supplement: Figure S2 — Q-Q plot for the p values achieved in the GWAS. (0.03 MB PDF) [file pgen.1000420.s002.pdf]

Figure S3

$R^2$  matrix

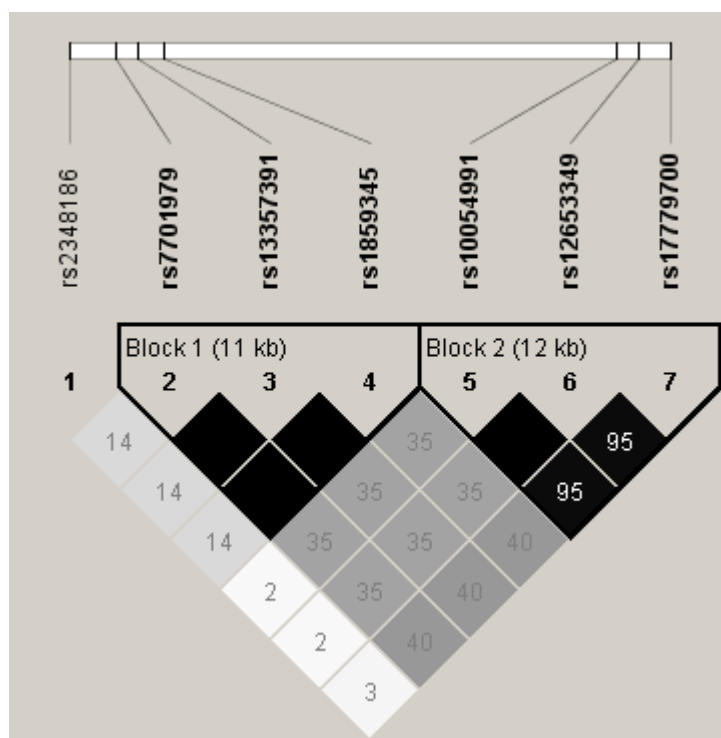

$D'$  matrix

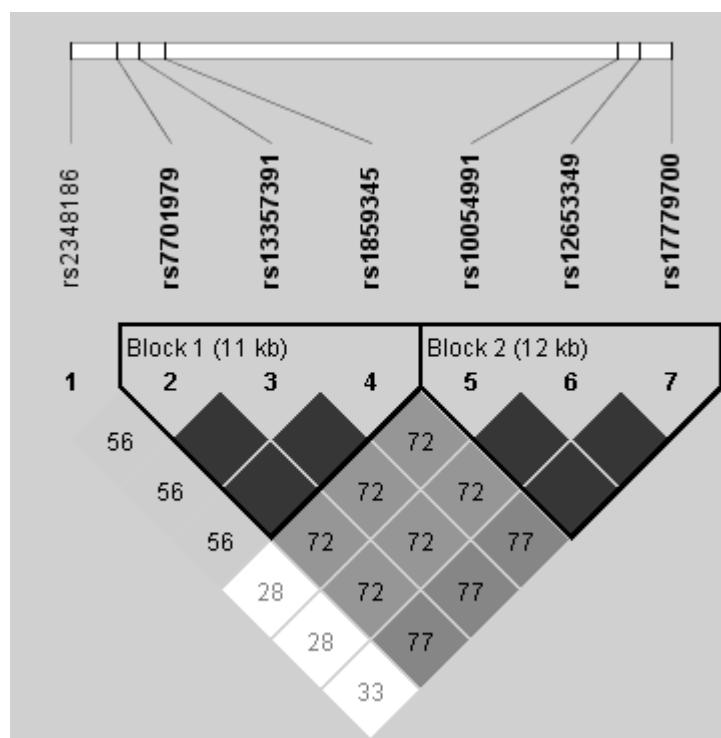

Supplement: Figure S3 — Haplotype block structure of the 7 AAM-associated SPOCK gene SNPs in Caucasians. The haplotype block structure was constructed using the HaploView software (http://www.broad.mit.edu/mpg/haploview/) [53] and the most recent SNP genotype data (HapMap Data Rel 26/phase III Nov 08, on NCBI B36 assembly, dbSNP b126) from HapMap (www.hapmap.org). The LD value between a certain pair of SNPs is shown within a corresponding “square”. A solid square without any value inside means a complete LD between the corresponding pair of SNPs. (0.03 MB PDF) [file pgen.1000420.s003.pdf]
