# Supplementary material for: Genome-Wide Association Analyses Identify SPOCK as a Key Novel Gene Underlying Age at Menarche
Source: PLoS Genet. 2009 Mar 13;5(3):e1000420. doi: 10.1371/journal.pgen.1000420 (PMC2652107; doi:10.1371/journal.pgen.1000420)
Supplement: Table S1 — SNPs of known genes that passed genome-wide significant FDR threshold (q<0.05) in the GWAS. (0.01 MB PDF) [file pgen.1000420.s004.pdf]

Table S1

SNP name	Associated genes	*p* value
rs2348186	***SPOCK***	4.92×10^{-07}
rs17779700	***SPOCK***	4.81×10^{-06}
rs13357391	***SPOCK***	5.77×10^{-06}
rs7701979	***SPOCK***	8.03×10^{-06}
rs10054991	***SPOCK***	1.20×10^{-05}
rs1859345	***SPOCK***	1.58×10^{-05}
rs12653349	***SPOCK***	1.61×10^{-05}
rs4464317	*ANTXR1*	5.80×10^{-06}
rs4384823	*ANTXR1*	8.26×10^{-06}
rs6833249	*BANK1*	1.02×10^{-05}
rs17539393	*DAPP1*	1.18×10^{-05}
rs9903371	*DCXR*	7.09×10^{-06}
rs10477387	*HTR4*	3.55×10^{-06}
rs7208728	*KCNJ2*	5.17×10^{-06}
rs11077530	*KCNJ2*	6.00×10^{-06}
rs4817359	*KRTAP11-1*	7.86×10^{-06}
rs2105494	*KRTAP11-1*	8.61×10^{-06}
rs1633490	*LRRN6C*	5.44×10^{-06}
rs4514489	*PDZRN4*	4.68×10^{-06}
rs6838858	*PRDM5*	1.42×10^{-05}
rs2150597	*RP11-54H7.1*	1.15×10^{-05}
