# Supplementary material for: Genome-Wide Association Analyses Identify SPOCK as a Key Novel Gene Underlying Age at Menarche
Source: PLoS Genet. 2009 Mar 13;5(3):e1000420. doi: 10.1371/journal.pgen.1000420 (PMC2652107; doi:10.1371/journal.pgen.1000420)
Supplement: Table S2 — SPOCK SNPs and the p values for their association with AAM. Labeled in bold are SNPs that are significant at the genome-wide FDR level of 0.05 (q<0.05). 1The second allele represents the minor allele of each locus. 2Minor allele frequency calculated in our Caucasian study subjects. 3Minor allele frequency reported for Caucasians in the public database of HapMap CEU. (0.03 MB PDF) [file pgen.1000420.s005.pdf]

Table S2

SNP	Name	Position	Role	Allele[1]	MAF[2]	MAF[3]	*p* value
1	*rs2429088*	136333359	3' UTR	A/C	0.126	0.117	0.105
2	*rs2429087*	136333565	3' UTR	C/T	0.130	0.117	0.060
3	*rs1051853*	136341700	Exon 11	G/T	0.134	0.083	0.161
4	*rs6871347*	136347268	Intron 9	A/T	0.404	0.433	0.295
5	*rs6872714*	136347755	Intron 9	A/G	0.403	0.432	0.302
6	*rs7722698*	136349312	Intron 8	C/G	0.134	0.108	0.051
7	*rs2347951*	136359122	Intron 6	C/T	0.494	0.483	0.447
8	*rs4308478*	136362213	Intron 6	A/G	0.089	0.092	0.554
9	*rs6863244*	136363847	Intron 6	C/T	0.464	0.467	0.847
10	*rs7719358*	136366264	Intron 6	A/G	0.269	0.308	0.451
11	*rs4434375*	136367046	Intron 6	G/T	0.121	0.108	0.196
12	*rs10067912*	136373568	Intron 6	A/G	0.120	0.108	0.200
13	*rs17521350*	136385265	Intron 6	A/G	0.044	0.050	0.268
14	*rs13167492*	136387218	Intron 6	C/T	0.123	0.117	0.037
15	*rs17646556*	136387395	Intron 6	A/G	0.124	0.110	0.050
16	*rs6899247*	136387845	Intron 6	A/G	0.197	0.181	0.031
17	*rs17521509*	136399410	Intron 6	C/T	0.396	0.367	0.256
18	*rs1468295*	136417494	Intron 6	A/T	0.215	0.161	0.018
19	*rs7721942*	136433496	Intron 5	A/G	0.215	0.258	0.013
20	*rs10434747*	136435929	Intron 5	A/G	0.449	0.417	0.179
21	*rs10039903*	136436236	Intron 5	A/G	0.230	0.258	0.025
22	*rs2189599*	136437484	Intron 5	A/G	0.202	0.142	0.004
23	*rs4976402*	136445259	Intron 5	A/T	0.310	0.292	0.305
24	*rs4976403*	136449090	Intron 5	C/T	0.018	0.008	0.701
**25**	***rs2348186***	**136451658**	**Intron 5**	**T/C**	**0.464**	**0.492**	**4.92×10^{-7}**
26	*rs13360974*	136459022	Intron 5	A/G	0.341	0.325	0.319
27	*rs6888621*	136459049	Intron 5	A/G	0.282	0.333	0.082
28	*rs17600276*	136461285	Intron 5	C/T	0.209	0.192	0.323
**29**	***rs7701979***	**136463382**	**Intron 5**	**G/T**	**0.367**	**0.308**	**8.03×10^{-6}**
**30**	***rs13357391***	**136468981**	**Intron 5**	**T/C**	**0.344**	**0.308**	**5.77×10^{-6}**
**31**	***rs1859345***	**136475319**	**Intron 5**	**T/C**	**0.343**	**0.308**	**1.58×10^{-5}**
32	*rs1859346*	136475473	Intron 5	A/C	0.318	0.300	1.00×10^{-4}
33	*rs6869576*	136477238	Intron 4	A/G	0.147	0.158	0.400
34	*rs2905965*	136482770	Intron 4	C/G	0.120	0.100	0.098
35	*rs2905967*	136484135	Intron 4	C/G	0.262	0.242	0.140
36	*rs2905968*	136485735	Intron 4	C/G	0.213	0.192	0.174
37	*rs916980*	136488540	Intron 4	C/T	0.434	0.492	0.146
38	*rs2905979*	136494603	Intron 4	C/T	0.120	0.100	0.088
39	*rs10050568*	136497195	Intron 4	A/C	0.258	0.242	0.289
40	*rs1434654*	136505991	Intron 3	A/G	0.123	0.102	0.133
41	*rs17776503*	136507420	Intron 3	C/T	0.137	0.142	0.777
42	*rs11746206*	136511813	Intron 3	A/G	0.142	0.133	0.783
43	*rs1434660*	136512738	Intron 3	A/C	0.445	0.483	0.282
44	*rs1434664*	136521719	Intron 3	A/C	0.121	0.093	0.020
45	*rs1434665*	136521738	Intron 3	A/T	0.301	0.258	3.21×10^{-5}
46	*rs2043478*	136526392	Intron 3	A/G	0.331	0.340	0.118
47	*rs11746562*	136527330	Intron 3	A/G	0.191	0.275	0.013

48	*rs9327774*	136533168	Intron 3	G/T	0.422	0.350	0.148
49	*rs17777250*	136538446	Intron 3	C/G	0.051	0.034	0.343
50	*rs7727317*	136538565	Intron 3	C/G	0.032	0.017	0.380
51	*rs10515489*	136538837	Intron 3	C/T	0.140	0.142	0.880
52	*rs4976416*	136551355	Intron 3	A/G	0.327	0.302	0.036
53	*rs17777965*	136560610	Intron 3	A/C	0.452	0.467	0.165
54	*rs892612*	136561264	Intron 3	G/T	0.128	0.150	0.496
55	*rs4435858*	136563465	Intron 3	A/T	0.151	0.158	0.209
56	*rs10064036*	136570066	Intron 3	C/T	0.451	0.467	0.184
57	*rs979666*	136579635	Intron 3	A/G	0.208	0.225	0.192
58	*rs2060425*	136581137	Intron 3	C/T	0.444	0.467	0.244
59	*rs6888866*	136586310	Intron 3	G/T	0.446	0.467	0.268
60	*rs6894483*	136586942	Intron 3	G/T	0.446	0.458	0.357
61	*rs6876013*	136587449	Intron 3	C/T	0.444	0.449	0.297
**62**	***rs10054991***	**136587711**	**Intron 3**	**A/G**	**0.235**	**0.233**	**1.20×10^{-5}**
63	*rs6865242*	136589045	Intron 3	A/C	0.040	0.017	0.044
64	*rs1865401*	136589671	Intron 3	A/C	0.322	0.308	0.053
65	*rs2916626*	136590483	Intron 3	G/T	0.041	0.033	0.040
**66**	***rs12653349***	**136593147**	**Intron 3**	**A/G**	**0.237**	**0.233**	**1.61×10^{-5}**
67	*rs6892706*	136599149	Intron 3	C/T	0.039	0.017	0.026
68	*rs10900848*	136599311	Intron 3	A/G	0.445	0.467	0.297
**69**	***rs17779700***	**136600692**	**Intron 3**	**A/G**	**0.233**	**0.208**	**4.81×10^{-6}**
70	*rs17780169*	136613498	Intron 3	A/G	0.138	0.150	0.570
71	*rs2961627*	136618045	Intron 3	C/T	0.194	0.233	0.086
72	*rs6867800*	136629902	Intron 3	C/T	0.461	0.475	0.144
73	*rs6596370*	136631080	Intron 2	A/G	0.361	0.358	0.010
74	*rs2961623*	136632149	Intron 2	C/T	0.336	0.350	0.219
75	*rs1434642*	136632772	Intron 2	C/T	0.040	0.017	0.007
76	*rs2060428*	136633415	Intron 2	C/T	0.333	0.350	0.263
77	*rs1434644*	136633984	Intron 2	C/T	0.336	0.350	0.185
78	*rs1434645*	136634212	Intron 2	C/T	0.343	0.339	0.228
79	*rs1434646*	136634243	Intron 2	C/T	0.334	0.350	0.317
80	*rs1434649*	136636576	Intron 2	A/G	0.336	0.350	0.274
81	*rs17171270*	136636619	Intron 2	C/T	0.038	0.017	0.010
82	*rs2961645*	136638021	Intron 2	A/G	0.141	0.169	0.028
83	*rs17781969*	136642926	Intron 2	A/G	0.136	0.150	0.617
84	*rs4976348*	136643294	Intron 2	C/T	0.194	0.175	0.001
85	*rs735824*	136643951	Intron 2	C/G	0.040	0.017	0.010
86	*rs988856*	136647417	Intron 2	A/G	0.137	0.150	0.616
87	*rs757128*	136648485	Intron 2	C/T	0.023	0.009	0.146
88	*rs2916624*	136648953	Intron 2	C/T	0.199	0.207	0.024
89	*rs988307*	136650744	Intron 2	A/G	0.368	0.342	0.402
90	*rs2916643*	136655146	Intron 2	A/G	0.133	0.169	0.018
91	*rs10515494*	136664924	Intron 2	C/T	0.044	0.017	0.042
92	*rs17782938*	136666217	Intron 2	C/T	0.134	0.150	0.363
93	*rs2916645*	136666248	Intron 2	C/T	0.149	0.181	0.037
94	*rs2961634*	136666679	Intron 2	A/G	0.154	0.172	0.068
95	*rs10053954*	136678744	Intron 2	A/G	0.235	0.225	0.102
96	*rs17522183*	136695950	Intron 2	A/G	0.205	0.175	0.285
97	*rs17171357*	136700884	Intron 2	A/G	0.038	0.025	0.022

98	*rs2348458*	136703268	Intron 2	C/T	0.039	0.025	0.031
99	*rs7732189*	136712418	Intron 2	A/G	0.460	0.483	0.027
100	*rs1229732*	136712667	Intron 2	A/G	0.407	0.458	0.111
101	*rs1229728*	136714733	Intron 2	C/T	0.405	0.458	0.107
102	*rs13180942*	136724076	Intron 2	A/G	0.152	0.150	0.469
103	*rs1229704*	136733607	Intron 2	A/G	0.058	0.042	0.297
104	*rs1229703*	136734283	Intron 2	G/T	0.254	0.217	0.324
105	*rs17171378*	136737893	Intron 2	C/G	0.013	0.000	0.800
106	*rs10479154*	136749046	Intron 2	A/T	0.025	0.008	0.124
107	*rs17171382*	136758512	Intron 2	A/T	0.144	0.117	0.342
108	*rs1229718*	136771691	Intron 2	A/G	0.352	0.367	0.060
109	*rs7719107*	136776756	Intron 2	A/G	0.018	0.008	0.112
110	*rs17171393*	136777467	Intron 2	C/T	0.018	0.008	0.111
111	*rs2141482*	136788377	Intron 2	C/T	0.038	0.025	0.008
112	*rs4246789*	136788702	Intron 2	C/T	0.018	0.008	0.111
113	*rs2950955*	136789291	Intron 2	A/C	0.354	0.367	0.058
114	*rs1105716*	136789997	Intron 2	C/T	0.019	0.008	0.111
115	*rs1105717*	136790089	Intron 2	A/G	0.017	0.008	0.112
116	*rs6596383*	136806718	Intron 2	A/G	0.254	0.283	0.001
117	*rs10434788*	136808389	Intron 2	G/T	0.463	0.450	0.019
118	*rs4246793*	136809940	Intron 2	C/T	0.277	0.300	1.80×10^{-4}
119	*rs17727364*	136815797	Intron 2	C/T	0.195	0.217	0.036
120	*rs4976445*	136820925	Intron 2	A/G	0.276	0.300	2.10×10^{-4}
121	*rs6882032*	136825989	Intron 2	A/C	0.243	0.242	0.001
122	*rs1528968*	136826340	Intron 2	A/C	0.460	0.450	0.016
123	*rs6596384*	136830446	Intron 2	C/T	0.265	0.267	0.003
124	*rs1528972*	136835625	Intron 2	C/T	0.262	0.267	0.003
125	*rs2348605*	136838003	Intron 2	C/G	0.454	0.450	0.022
126	*rs2974499*	136841710	Intron 2	C/G	0.386	0.400	0.517
127	*rs2141481*	136848474	Intron 2	A/T	0.364	0.342	0.064
128	*rs1881620*	136852560	Intron 2	C/T	0.364	0.353	0.062
129	*rs4976450*	136856221	Intron 2	A/G	0.361	0.347	0.062
130	*rs10060914*	136861259	Intron 2	A/G	0.370	0.358	0.084
