# Supplementary material for: Genome-Wide Association Analyses Identify SPOCK as a Key Novel Gene Underlying Age at Menarche
Source: PLoS Genet. 2009 Mar 13;5(3):e1000420. doi: 10.1371/journal.pgen.1000420 (PMC2652107; doi:10.1371/journal.pgen.1000420)
Supplement: Table S3 — Conditional association analysis of the 7 AAM-associated SPOCK gene SNPs in the GWAS cohort. Block 1 and block 2 are the haplotype blocks as shown in Figure S3. Original p values are the p values achieved in regular association analysis for AAM. P values for some SNPs cannot be estimated due to the high LD between the SNPs and the SNP used as the covariate for conditional analysis. (0.02 MB PDF) [file pgen.1000420.s006.pdf]

Table S3

**SNP**	**Haplotype block**	**Original *p* values**	**Association *p* values conditioned on *rs2348186***	**Association *p* values conditioned on *rs13357391***	**Association *p* values conditioned on *rs12653349***
rs2348186	–	4.92×10^{-7}	–	4.65×10^{-4}	2.11×10^{-5}
rs7701979	Block 1	8.03×10^{-6}	0.019	–	9.23×10^{-3}
rs13357391	Block 1	5.77×10^{-6}	0.016	–	9.70×10^{-3}
rs1859345	Block 1	1.58×10^{-5}	0.024	–	1.61×10^{-3}
rs10054991	Block 2	1.20×10^{-5}	2.07×10^{-3}	0.033	–
rs12653349	Block 2	1.61×10^{-5}	2.07×10^{-3}	0.033	–
rs17779700	Block 2	4.81×10^{-6}	1.88×10^{-3}	0.029	0.755
